# Ubiquitin like protein FAT10 repressed cardiac fibrosis after myocardial ischemic via mediating degradation of Smad3 dependent on FAT10-proteasome system

**DOI:** 10.7150/ijbs.77677

**Published:** 2023-01-16

**Authors:** Chen Chen, Xiaoqing Li, Tao Zhou, Yuhao Su, Bodong Yu, Jiejing Jin, Jinyan Xie, Yang Shen, Rong Wan, Kui Hong

**Affiliations:** 1 Department of Cardiovascular Medicine, the Second Affiliated Hospital of Nanchang University, Nanchang of Jiangxi, 330006 China; 2 Jiangxi Key Laboratory of Molecular Medicine, Nanchang of Jiangxi, 330006 China; 3 Department of Genetic Medicine, the Second Affiliated Hospital of Nanchang University, Nanchang of Jiangxi, 330006 China; 4 Second College of Clinical Medicine, Nanchang University, Nanchang, Jiangxi, 330006 China

**Keywords:** FAT10, Cardiac fibrosis, Myocardial infarction, Smad3, HiPSC-CFs

## Abstract

Cardiac fibrosis after myocardial ischemic (MI) injury is a key factor in heart function deterioration. We recently showed that ubiquitin-like protein human HLA-F adjacent transcript (FAT10) plays a novel role in ischemic cardiovascular diseases, but its function in cardiac fibrosis remains unknown. The present study aims to detail the pathophysiological function of FAT10 in MI injury-induced cardiac fibrosis and its underlying mechanism. *In vivo*, a systemic FAT10 deficiency mouse (*Fat10*^-/-^) model was established which exhibited excessive cardiac fibrosis and deleterious cardiac function after MI when compared to wild-type mice. Cardiac fibrotic-related proteins (α-SMA, collagen I and collagen III) content were increased in MI-*Fat10*^-/-^ mice. Similarly, cardiac FAT10 restoration in *Fat10^-/-^* mice suppressed fibrosis and improved cardiac function. *In vitro*, FAT10 overexpression exert a protective effect against the transforming growth β1 (TGF-β1)-induced proliferation, migration and differentiation in cardiac fibroblast (CFs), primary CFs from *Fat10^-/-^* mice and human induced pluripotent stem cell-derived CFs (hiPSC-CFs). Furthermore, immunoprecipitation-mass spectrometry (IP-MS) data demonstrated that FAT10 might mediate Smad3, a critical factor in cardiac fibrosis. Combined with rescue assays both *in vivo and vitro*, the protective effects of FAT10 against cardiac fibrosis was detected to be dependent on Smad3. In depth, Smad3 as a FAT10 specific substrate, FAT10 specifically bind to the K378 site of Smad3 directly via its C-terminal glycine residues and mediated the degradation of Smad3 through the FAT10-proteasome system instead of ubiquitin. In conclusion, we here show that FAT10 is a novel regulator against cardiac fibrosis after MI by mediating Smad3 degradation through FAT10-mediated proteasome system. Our study confirms the cardioprotective role of FAT10 in the heart, and providing a new prospective insight into the regulation of cardiac fibrosis after MI.

## Introduction

Myocardial ischemic (MI) is one of the main causes of death worldwide 1, 2. After MI, cardiac fibrosis is a pivotal pathological outcome which causes heart remodeling and heart failure 3, 4. Nevertheless, there is no specific drug for myocardial fibrosis. Recently, numerous researches have noted the activity of ubiquitin and ubiquitin-like proteins (UBLs) on MI-induced cardiac fibrosis. Inhibition of the ubiquitin-proteasome system has been found to interfere with collagen and matrix metalloproteinase expression 5. For instance, the E3 ubiquitin ligase WWP2 -regulated pro-fibrotic gene network could be conserved across different cardiac diseases characterized by fibrosis 6. Furthermore, inhibition of small ubiquitin-like modifier (SUMO1), one UBL family peptide, alleviated MI-induced heart dysfunction and fibrosis 7. However, whether another UBL family member, human HLA-F adjacent transcript (FAT10), affects fibrosis is still unknown.

FAT10 is a well-known modulator for proteasomal degradation. Lots of studies have focused on FAT10 in tumors, the cell cycle, apoptosis, and immune response 8. Till now, few studies have demonstrated the biological functions of FAT10 in the heart. In our previous studies, the protective roles of FAT10 in MI injury and its complication diseases were determined 9-11. First, FAT10 was showed to be expressed in heart. Then, the functions of FAT10 against myocardial injury-induced apoptosis were found not only be dependent by p53-mediated micro-143a pathway but also by stabilization of Cav3 9. In addition, further study relieved FAT10 repressed autophagy and protected the heart from MI-accompanied arrhythmia 12. However, whether FAT10 acts in the development of cardiac fibrosis after MI is unclear.

Transforming growth factor (TGF)-β1/Smads is a key fibrogenic growth signal in cardiac fibrosis 13, 14. giving the CFs the ability to proliferate, migrate, and synthesize collagen. Smad3, a member of the Smads family proteins, participates in TGF-β superfamily modulated signaling. In the fibrogenesis process, Smad3 is overactivated and subsequently bind to many collagen matrix promoters directly 15. Mice lacking Smad3 are protected against fibrosis in many cardiovascular diseases 16-19. Hence, the direct inhibition of Smad3 is a potential therapeutic target for cardiac fibrosis. Interestingly, studies have demonstrated that the stability of Smad3 is regulated by ubiquitination 20. In addition, the E3 ligase SMURF2 is proved to promote Smad3 ubiquitination 21. Nevertheless, it remains indefinite how UBL protein FAT10 affects the expression of Smad3 in ischemic-induced cardiac fibrosis.

In the current study, we investigated the functions and a novel underlying mechanism of FAT10 in ischemic-induced cardiac fibrosis *in vitro* and *in vivo*. The data supported that FAT10 bond to the K378 modification site of Smad3 and mediated its degradation via the FAT10-mediated proteasome system (FPS) which ultimately inhibited ischemic-induced cardiac fibrosis.

## Material and methods

Please see Supplementary material online for detailed Methods section.

### Mice

All animal care procedures and experiments were approved by the Animal Ethics and Experimentation Committee of Nanchang University and conformed to the “Guide for the Care and Use of Laboratory Animals”. Global FAT10 knockout (*Fat10^-/-^*) mice on a C57BL/6J background were purchased by Model Animal Research Center of Nanjing University. The detail information about *Fat10^-/-^*mice were shown in Supplementary material. The* Fat10*^-/-^ mice and wild-type (WT) control littermates were maintained on 12-hour light, 12-hour dark schedule with access to rodent lab chow and water. The mice were maintained according to the Guidelines for the Care and Use of Laboratory Animals formulated by the Ministry of Science and Technology of China. At the end of the experiment, all mice were anaesthetized by inhaling 2% isofluranein 100% oxygen (0.8-1.2 L/min) for 10 min, and then under cervical disarticulation.

### Cell culture

Neonatal rat cardiac fibroblasts (NRCFs) and primary mice CFs were isolated as described previously 34, 35. Cells were cultured in Dulbecco's modified Eagle's medium containing 10% fetal bovine serum. Human induced pluripotent stem cell-derived cardiac fibroblasts (hiPSC-CFs) were obtained from Help Regenerative Medicine Technology Co., Ltd. (Nanjing, China) and cultured in Dulbecco's modified Eagle's medium (Gibco®, Gaithersburg, MD, USA) containing 10% fetal bovine serum. The generation of *CRISPR-Cas9* knockout HEK293 cell lines was previously reported.^36^ All the cells were cultured at 37 °C and 5% CO2 incubator in a humidified atmosphere.

### Myocardial infarction surgery and echocardiography analysis

All adult male (8-weeks) mice were randomly used to performed myocardial infarction (MI) surgery by ligating the left anterior descending coronary artery as described previously.^12^ No significant fibrotic changes were observed in liver, lung, and kidney between Fat10-/- and WT sham-operated or MI mice group (Supplementary Fig. 2). Cardiac size and function were assessed by echocardiography as published (*Vevo 2100; Visual Sonics, Canada*) when animals were anaesthetized by isoflurane inhalation (2.5%) plus 1 L/min O2.

### Virus injection procedures

Recombinant adenovirus expressing FAT10 (*ad-Fat10*, 8*108 pfu/ml) was injected at a final volume of 9 μL into the hearts of *wild-type* and *Fat10*^-/-^consistently at three different points, ad-con as the respective negative controls. MI+*ad-con*/*ad-Fat10* refers to the mice that received virus injection and underwent LAD ligation, while sham+*ad-con*/*ad-Fat10* refers to the mice that received virus injection but did not undergo LAD ligation.

### Protein Identification by In-Gel Digestion and Mass Spectrometry

Proteins were extracted from border ischemic areas. The stained protein band was cut and then analyzed by immunoprecipitation-mass spectrometry (IP-MS) with the help of Novogene (Beijing Co., Ltd), who provided technological assistance. The MS data were analyzed using Q ExactiveTM series mass spectrometer (*Thermo Fisher*), with ion source of Nanospray Flex™ (*ESI*).

### Bimolecular fluorescence complementation (BiFC) assay

The BiFC assay was performed with modified protocol as described previously 37. Here, we fused FAT10 to the N-terminus of fluorescent protein Venus (*VN-Fat10*) and Smad3 to the C-terminus of fluorescent protein Venus (*VC-Smad3*), then transient transfected into HEK293 cells. The cells were fixed by 4% paraformaldehyde 48 hours after transfection. The fluorescence was observed by Leica laser microscope.

### Statistical Analysis

Statistical analysis was performed using GraphPad Prism version 8.0. The results were expressed as mean ± SEM for experiments conducted at least in triplicates. Unpaired t test was used for comparison between two groups. For comparisons among three or more sets of data with one factor or two factor, one-way or two-way ANOVA was used. P value less than 0.05 was considered significantly different.

## Results

### FAT10 deficiency aggravated ischemic-induced cardiac fibrosis *in vivo*

**Fig 1:**
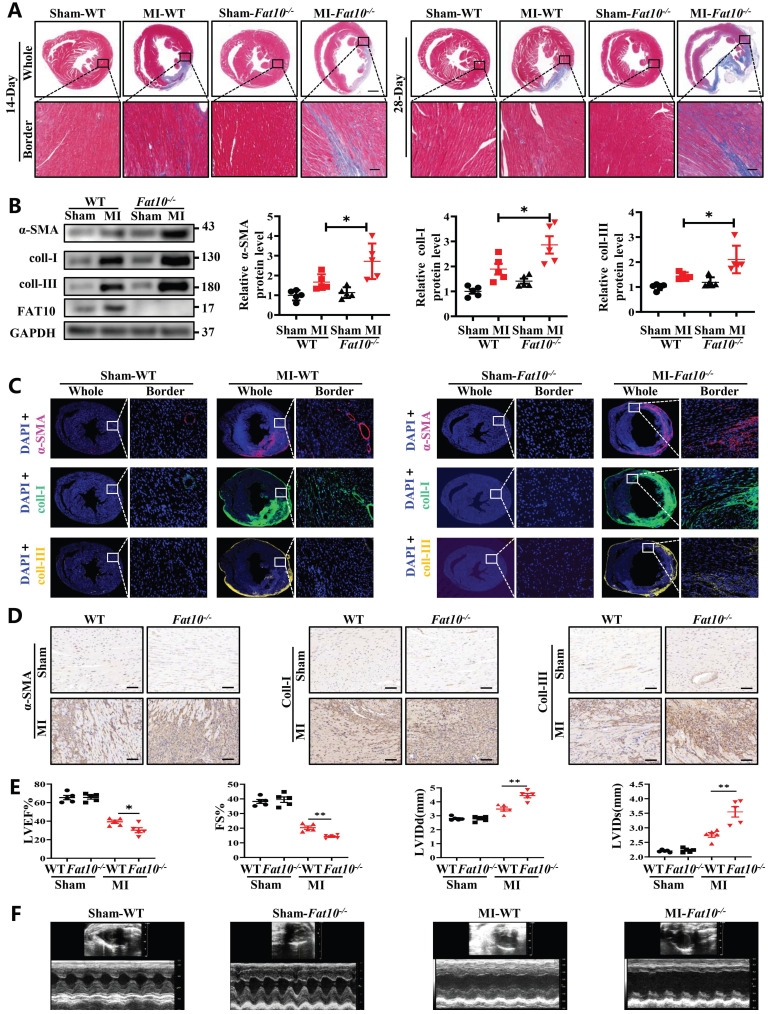
** FAT10 deficient mice exhibit increased cardiac fibrotic after myocardial infraction. A** Masson staining of whole heart fibrosis (up, bar 1mm) and infarct border fibrosis (down, bar 50μm) (n=4 per group) in 14-days and 28-days.** B** Western blot analysis of α-SMA, collagen I and collagen III in heart tissue from 14day-MI-*FAT10^-/-^* and 14day-MI-WT (n=5 per group). **C** Immunofluoresence staining of myocardial sections showing α-SMA (in red), collagen I (in green) and collagen III (in yellow) in 14day-MI-*FAT10^-/-^* and 14day-MI-*wild type* mice (n=4 per group). Nuclei were counterstained with DAPI (blue). **D** IHC staining of α-SMA, collagen I and collagen III of heart tissue from 14day-MI-*FAT10^-/-^* and 14day-MI-*wild-type* (bar 50μm) (n=4 per group). **E** Echocardiographic analysis to evaluate left ventricular ejection fraction (LVEF), fractional shortening (FS), left ventricular internal diameter at end-diastole (LVIDd) and left ventricular internal diameter at end-systole (LVIDs) in WT and *Fat10*^-/-^ after sham and 2 weeks post-MI surgery (n=5 per group). **F** Representative M-mode echocardiographic images of WT and *Fat10*^-/-^ hearts. *p<0.05, **p<0.01, ***p<0.001 and ns=not significant. All data presented as mean±SEM.

To explore the effects of FAT10 on heart under ischemic condition, *Fat10^-/-^* mice were established first and the expressions of FAT10 in heart were examined (Supplementary Fig. 1A). In addition, no difference in the basic weight growth as well as the baseline of cardiac function of *Fat10^-/-^* mice in 8 weeks were detected (Supplementary Fig. 1B and Supplementary Table. 1). Then, the* Fat10^-/-^* mice were subjected to MI surgery along with the littermate WT mice (the control group). First, the degree of cardiac fibrosis in different times (7 days, 14days and 28days) after MI between *Fat10^-/-^* and WT mice were detected. As shown in Fig. 1A and Supplementary Fig. 3A-B, Masson staining and Sirius Red staining revealed a highly increased fibrotic area both in whole heart and border zone in MI-*Fat10^-/-^* group mice hearts. Meanwhile, western blotting showed that the expressions of typical fibrosis-related proteins (α-SMA, collagen I and collagen III) were increased in the hearts of MI-*Fat10^-/-^* (Fig. 1B). In addition, immunofluorescence, and immunocytochemistry (IHC) assay demonstrated that fibrosis-related proteins were all upregulated in the MI-*Fat10^-/-^* group compared with those in MI-WT group (Fig. 1C, D; Supplementary Fig.3C-3D). Furthermore, echocardiographic analysis was conducted to detect the cardiac function after MI. In sham-operated mice, no significant changes were observed in the left ventricular ejection fraction (LVEF), fraction shorting (FS), left ventricular internal diameter at end-diastole (LVIDd) and left ventricular internal diameter at end-systole (LVIDs) between MI-WT and MI-*Fat10^-/-^* groups. After MI surgery, LVEF and FS were severely reduced, LVIDd and LVIDs were obviously increased in the MI-*Fat10^-/-^* group (Fig. 1E, F). Finally, consistent with deteriorated cardiac function, increased mortality (Supplementary Fig.3E) was also observed in MI-*Fat10^-/-^* group. Taken together, these results strongly suggest that FAT10 deletion is contributable in cardiac fibrosis following MI.

### Overexpression of FAT10 in mice heart alleviated the ischemic-induced cardiac fibrosis

**Fig 2:**
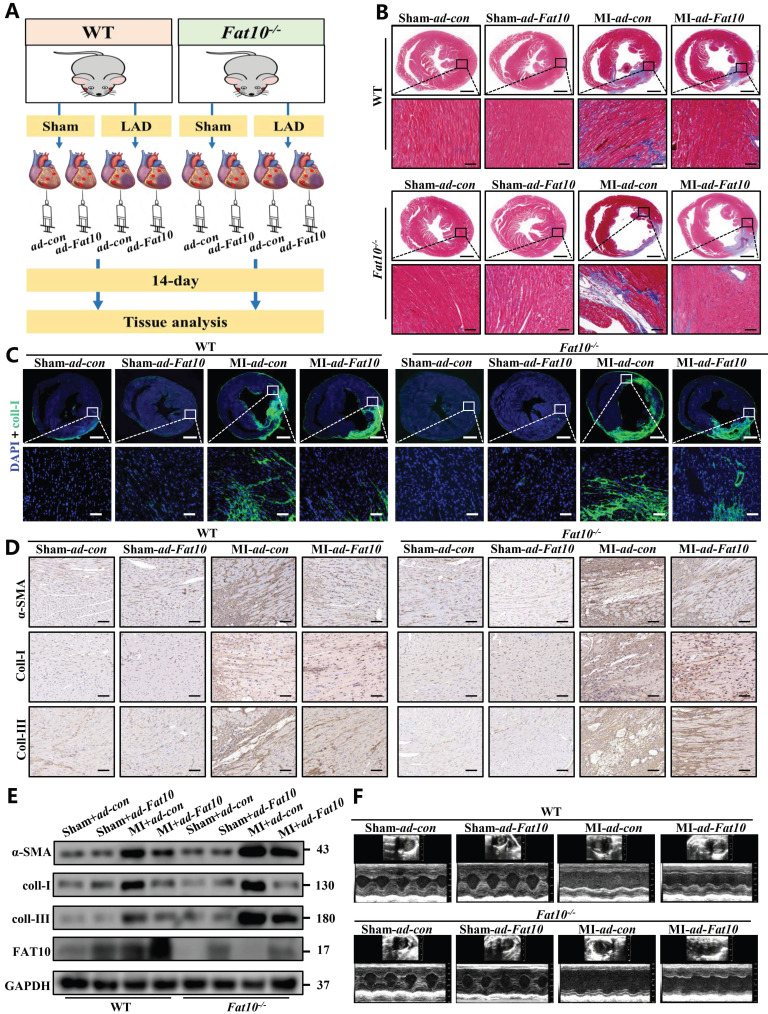
** FAT10 overexpression alleviated MI-induced cardiac fibrosis. A** Experimental procedure for injecting mice with *ad-Fat10* virus*.* **B** Masson stainin of whole heart fibrosis (bar 1mm) and infarct border fibrosis (bar 50μm) (n=6 per group). **C** Immunofluoresence staining of myocardial sections showing collagen I (in green). Nuclei were counterstained with DAPI (blue). **D** IHC staining of α-SMA, collagen I and collagen III of heart tissue from per group. **E** Western blot analysis of α-SMA, collagen I and collagen III in heart tissue from per group. **F** Representative M-mode echocardiographic images. *p<0.05, **p<0.01, ***p<0.001 and ns=not significant. All data presented as mean±SEM.

Since severe cardiac fibrosis was observed in *Fat10^-/-^* mice after MI, to confirm the protective effects of FAT10 against cardiac fibrosis after MI, recombinant adenovirus expressing FAT10 were used in *Fat10^-/-^*mice heart tissue by vector infection, and mice subjected to MI surgery were observed for 14 days (Fig. 2A). The efficiency of FAT10 overexpression was assessed by western blotting (Supplementary Fig. 4A). As shown in Fig. 2B, MI surgery dramatically increased fibrosis in the hearts of the *Fat10^-/-^* group, whereas these changes were rescued in the *ad-Fat10* group, the quantification of the fibrotic area were shown in Supplementary Fig. 4B. Western blotting, immunofluorescence, and IHC staining also showed that *ad-Fat10* injection was associated with diminished expressions of fibrotic markers (Fig. 2C-E). Next, restoration of FAT10 expression in *Fat10^-/-^* mice also improved cardiac function, as detected by echocardiography (Fig. 2F; Supplementary Fig.4 D-G). Therefore, these findings suggest that overexpression of FAT10 may exert a beneficial effect on cardiac fibrosis under MI.

### FAT10 reversed TGF-β1-induced fibrotic response in *vitro*

**Fig 3:**
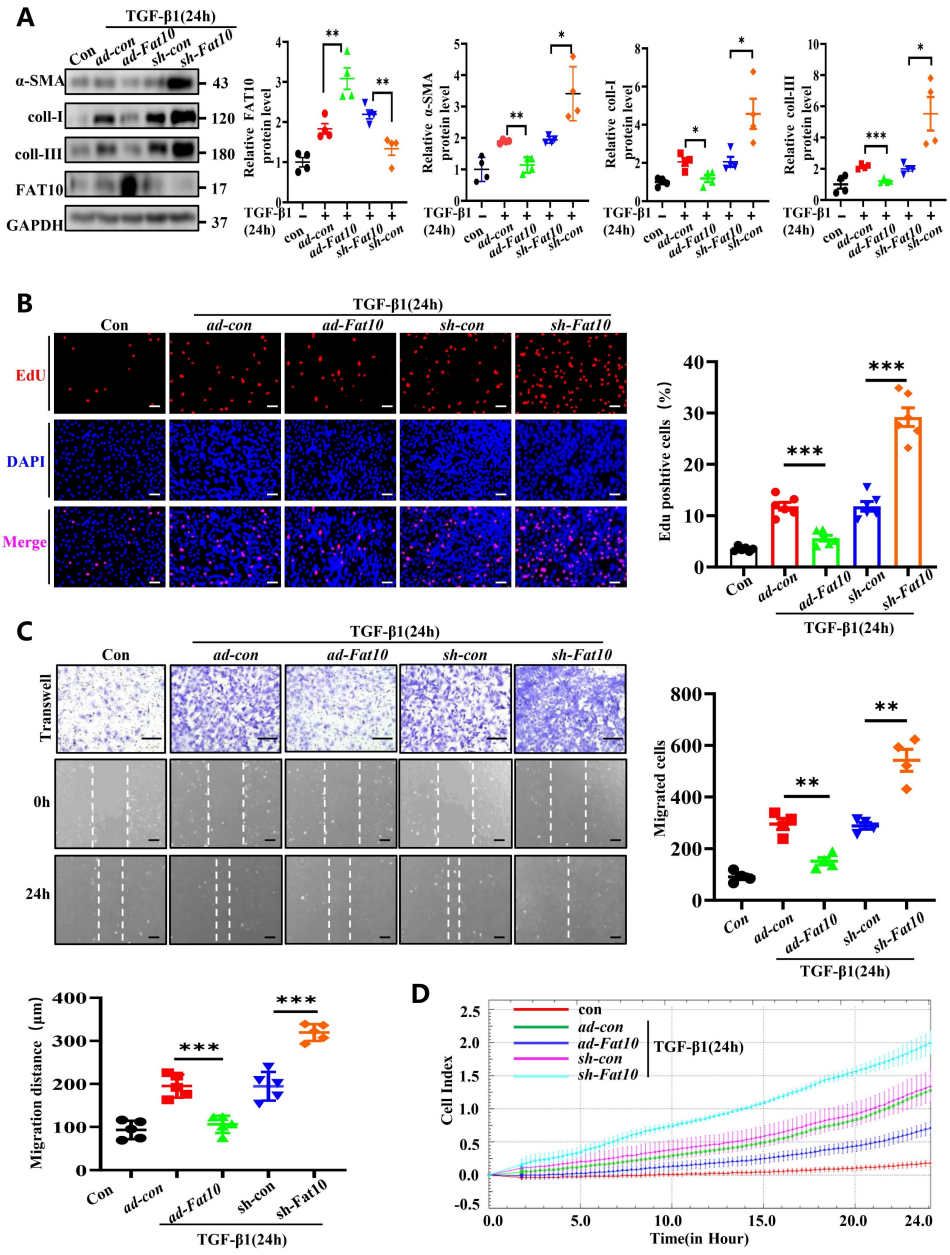
** FAT10 negatively regulates the TGF-β1-induced fibrosis in vitro. A** NRCFs were transfected with *ad-Fat10* or* sh-Fat10* and then subjected to treated with TGF-β1(10ng/ml), the level of α-SMA, collagen I and collagen III were detected by western blot analysis (n=4 per group). **B** Representative immunofluorescence images of EdU(red) and DAPI (blue)(left) and quantitative data (right) in the different experimental groups of NRCFs (bar 50μm) (n=6 per group). **C** Migration of NRCFs measured by Transwell assay (bar 100μm) (n=4 per group) and wound-healing assay (n=5 per group) (bar 100μm). Statistical results of relative migration of speed were analyzed (n=4 per group). **D** Migration of NRCFs measured by RTCA assay. *p<0.05, **p<0.01, ***p<0.001 and ns=not significant. All data presented as mean±SEM.

TGF-β1, a reported classic regulator in the progression of fibrosis after MI, can acquire fibrotic phenotypes 22. Accordingly, NRCFs were treated with 10 ng/mL TGF-β1 for 24h to establish fibrosis model. To determine whether FAT10 modulates cardiac fibrosis *in vitro*, cells were transfected with *ad-Fat10* and *sh-Fat1*0 viruses before TGF-β1 treatment. Data from the western blotting assay revealed that FAT10 overexpression markedly inhibited the TGF-β1-induced increase in fibrosis-related proteins (α-SMA, collagen I, and collagen III) in NRCFs, whereas FAT10 knockdown showed the opposite effect (Fig. 3A). Then, the major characteristics of fibrosis, including cell differentiation, collagen synthesis, proliferation, and migration, were examined. The cell proliferation ratio of NRCFs significantly decreased when FAT10 was overexpressed, as detected by EdU staining, but increased when FAT10 was deleted (Fig. 3B). Finally, as shown in Fig. 3C-D, upregulation of FAT10 significantly suppressed TGF-β1-induced cell migration, whereas inhibition of FAT10 showed the opposite effect by wound healing and RTCA assays.

Consistent with the above results in NRCFs, primary mouse CFs from *Fat10*^-/-^ mice showed increased cell differentiation, collagen synthesis, cell proliferation, and migration compared with WT-CFs after treatment with TGF-β1; these effects were reversed with FAT10 restoration (Supplementary Fig. 5). Taken together, our results indicate that FAT10 inhibits TGF-β1-induced fibrotic CFs and exerts a markable anti-fibrotic effect on cardiac fibrosis.

### Smad3 is a novel FAT10 target substrate and plays a critical role of FAT10 regulating cardiac fibrosis after MI

**Fig 4:**
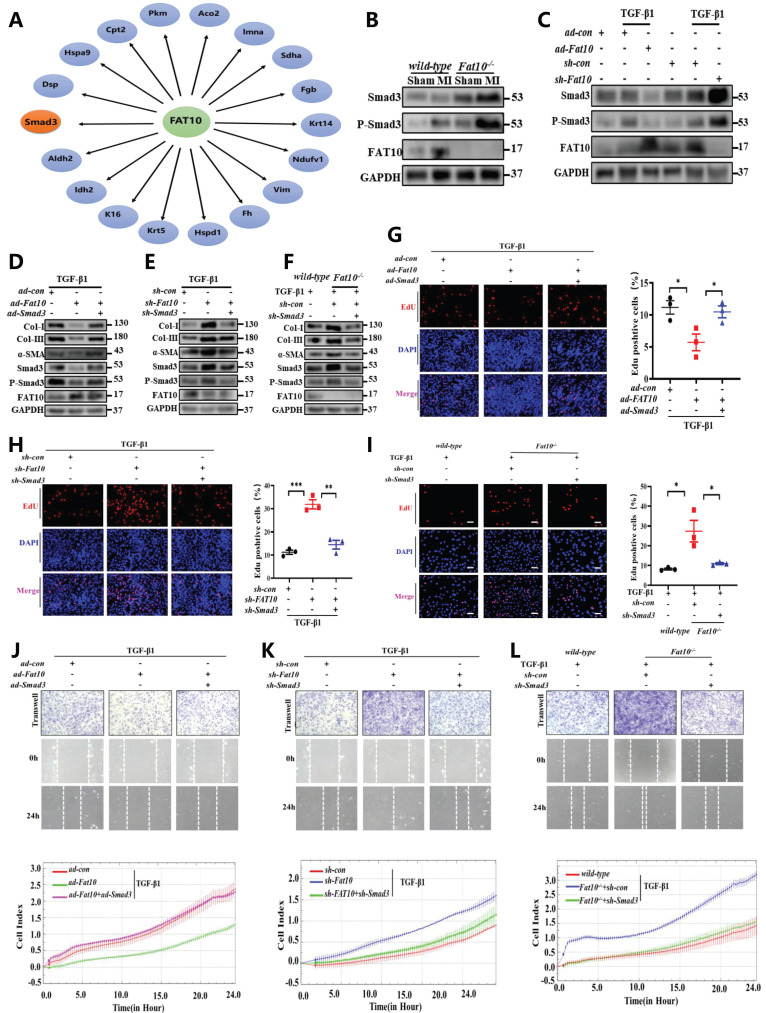
** FAT10 regulates MI-induced cardiac fibrosis by targeting Smad3. A** The interaction of FAT10 and its potential substrates.** B** Western blot analysis of Smad3 and P-Smad3 in heart tissue from MI-*Fat10*^-/-^ and MI-WT. **C** NRCFs were transfected with *ad-Fat10* or *sh-Fat10* and then exposed to TGF-β1, the expression of Smad3 and P-Smad3 was measured by western blot analysis. **D** NRCFs were co-transfected with *ad-Fat10* and *ad-Smad3* or *ad-con* and then exposed to TGF-β1; α-SMA, Smad3, P-Smad3, collagen I and collagen III were detected by western blot analysis. **E** NRCFs were co-transfected with *sh-Fat10* and *sh-Smad3* or *sh-con* and then exposed to TGF-β1; α-SMA, Smad3, P-Smad3, collagen I and collagen III were detected by western blot analysis. **F** *Fat10*^-/-^ and WT primary CF were transfected with *sh-Smad3* then exposed to TGF-β1. α-SMA, Smad3, P-Smad3, collagen I and collagen III were detected by western blot analysis. **G-I** Representative immunofluorescence images of EdU (red) and DAPI (blue)(left) and quantitative data (right) in the different experimental groups of NRCFs (n=3 per group) (bar 50μm).** J-L** Migration of NRCFs measured by Transwell assay (bar 100μm), wound-healing assay (bar 100μm) and RTCA assay. *p<0.05, **p<0.01, ***p<0.001 and ns=not significant. All data presented as mean±SEM.

To explore the further molecular mechanism of FAT10 on cardiac fibrosis, immunoprecipitation-mass spectrometry (IP-MS) analysis was carried out to screen the potential interacting proteins of FAT10. Indeed, 195 peptides were identified as the possible FAT10 substrates. Among the candidate FAT10-associated target proteins, 18 peptides were reportedly to be involved in fibrosis as listed in Fig. 4A. Combined the above data from IP-MS and the publication by Yifat et al. 23, it should be noticed that Smad3 is the only protein that may be involved in fibrosis as a FAT10 substrate. Furthermore, Smad3 plays a critical role in cardiac fibrosis. Therefore, we hypothesised that Smad3 is the downstream molecular factor of FAT10 and participates in* Fat10^-/-^*-induced cardiac fibrosis. To test this hypothesis, we first evaluated the effect of FAT10 on Smad3 expression. Western blotting results revealed that the expression of Smad3 was higher in the heart tissues of MI-*Fat10*^-/-^ mice than in MI-WT mice (Fig. 4B). This was consistent with the data from in vivo studies. As shown in Fig. 4C, NRCFs treated with* ad-Fat10* showed decreased Smad3 expressions, whereas suppression of FAT10 increased Smad3 expressions. Indeed, we found that FAT10 only affected Smad3 expression, but failed to change Smad2 or Smad4expressions (Supplementary Fig. 6).

**Fig 5:**
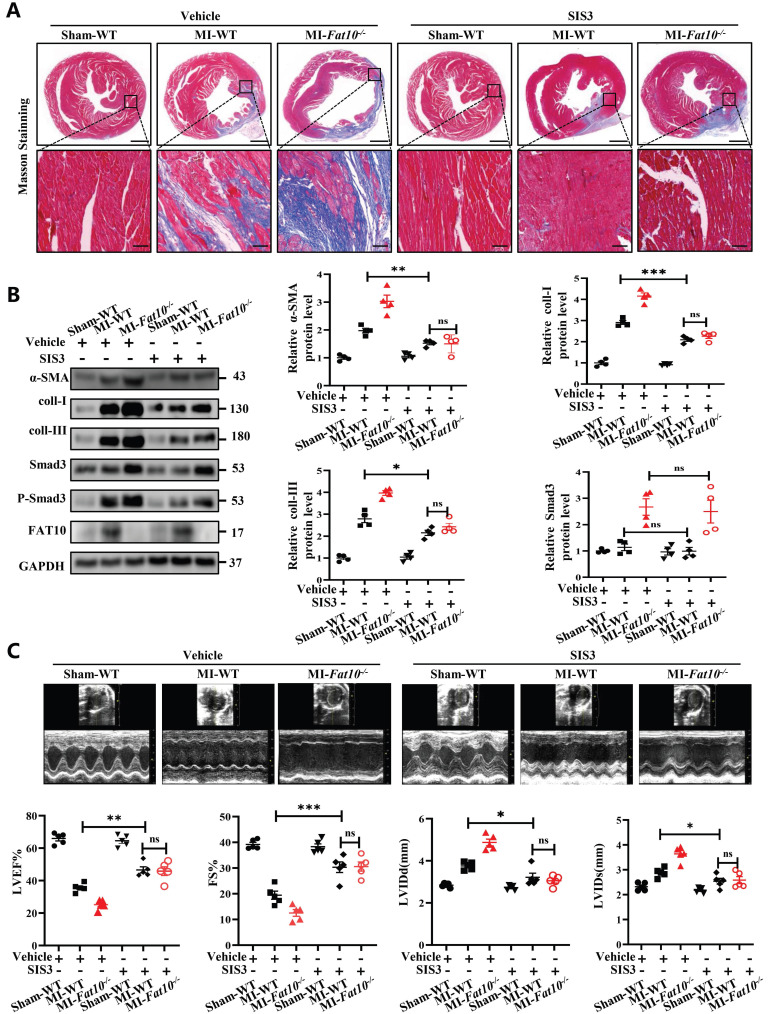
** Smad3-specific inhibitor (SIS3) inhibited *Fat10^-/-^*-induced cardiac fibrosis after MI. A** Masson staining of whole heart fibrosis (bar 1mm) and infarct border fibrosis (bar 50μm) (n=4 per group). **B** Western blot analysis of α-SMA, collagen I and collagen III in heart tissue from per group (n=4 per group). **C** Echocardiographic analysis to evaluate LVEF, FS, LVIDd and LVIDs and representative M-mode echocardiographic images (n=5 per group). *p<0.05, **p<0.01, ***p<0.001 and ns=not significant. All data presented as mean±SEM.

To further investigate a causative relationship between Smad3 upregulation and exacerbated MI-induced cardiac fibrosis, a Smad3-specific inhibitor (SIS3) were administered daily to mice (5mg/kg/day), beginning on the first day after MI injury. As shown in Fig. 5A and Supplementary Fig.S9, consistent with the above results, compared with the WT-MI+vehicle group, the Fat10^-/-^ MI+vehicle group showed deteriorated cardiac fibrosis and function. Then, the efficiency of SIS3 on cardiac fibrosis was also assessed. Our results showed that compared with WT, the SIS3 administration was associated with diminished fibrosis (Fig. 5A), decreased expression of fibrotic markers (Fig. 5B), and improved cardiac function (Fig. 5C). Importantly, our results demonstrated that when mice were treated with SIS3, FAT10 deficiency can't aggravate MI-induced cardiac fibrosis (Fig.5A-C), which means that the aggravation of fibrosis after MI caused by FAT10 knockout was blocked upon the administration of SIS3. Taken together, these findings suggest that Smad3 mediates *Fat10^-/-^* induced fibrosis after MI *in vivo*.

Subsequently, in NRCFs, as shown in Fig. 4D and Supplementary Fig. 7A, the expressions of fibrosis-related proteins were reduced after transfection with *ad-Fat10*, however, this change was significantly abolished when Smad3 was restored by *ad-Smad3*. Similarly, Smad3 inhibition blocked the increase of fibrosis markers induced by FAT10 deficiency (Fig. 4E; Supplementary Fig. 7B;). Consistently, the same effect was observed in proliferation (Fig. 4G, H) and migration (Fig. 4J, K; Supplementary Fig. 8A-B) experiments. Moreover, we used *Fat10*^-/-^ mouse primary CFs to further confirm that FAT10 knockdown induced upregulation of the fibrotic response was significantly abolished by* sh-Smad3* (Fig. 4F, I and L; Supplementary Fig. 7C; Supplementary Fig. 8C).

### FAT10 promoted Smad3 degradation through FAT10-proteasome system (FPS)

**Fig 6:**
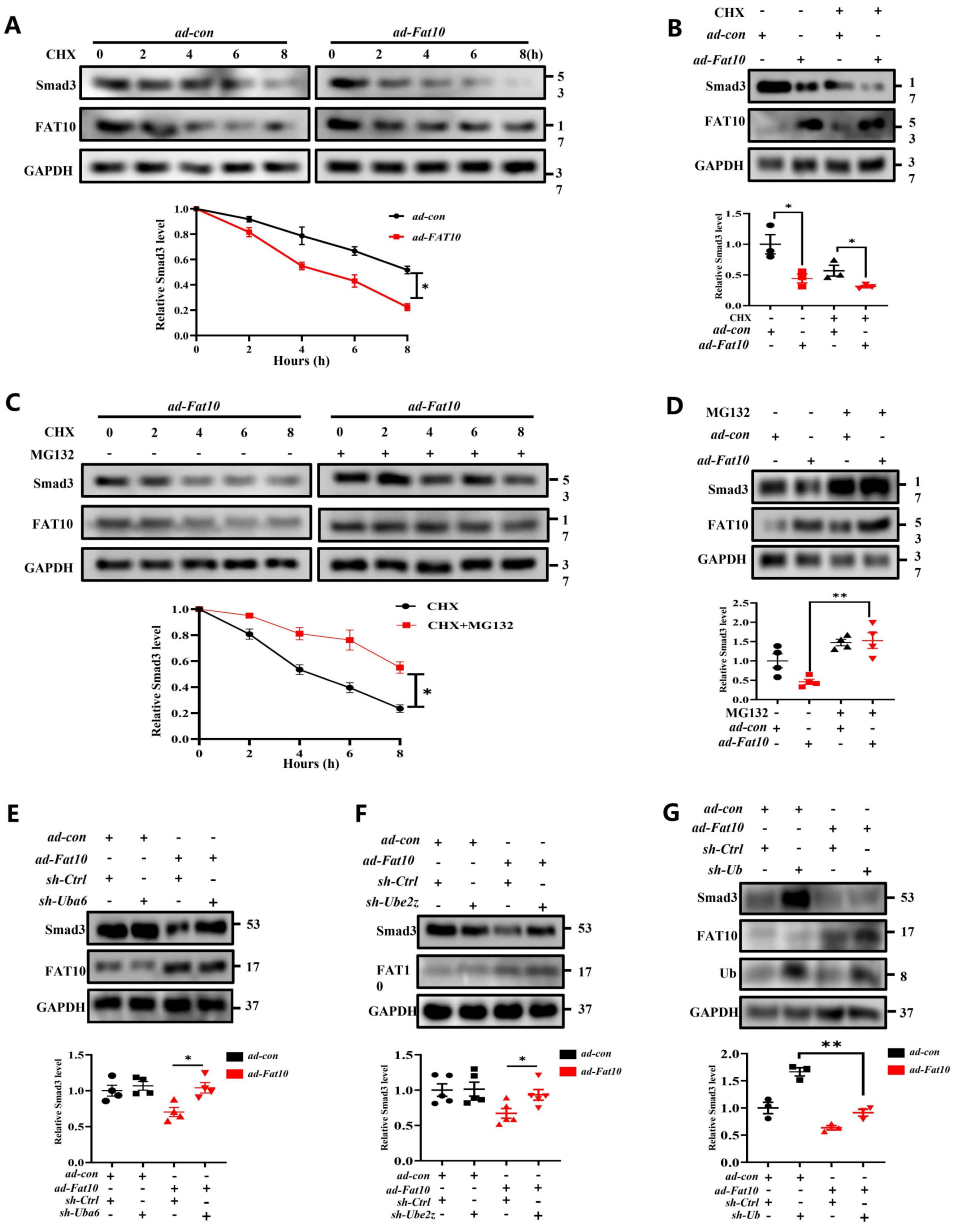
** Smad3 plays key role of FAT10 regulating cardiac fibrosis after MI. A** NRCFs were treated with CHX (20 μM) for the indicated times in the presence or absence of* ad-Fat10*, and the levels of Smad3 were then detected. **B** NRCFs were transfected with *ad-Fat10*, the cells were then exposed to CHX (20 μM), and the Smad3 levels in the supernatants were measured by western blot. **C** NRCFs were treated with CHX (20 μM) for the indicated times in the presence or absence of MG132 (15 μM), and the levels of Smad3 were then detected (n=3 per group). **D** Cells in each group were treated with MG132 (15 μM) for 12 h, and the expression of Smad3 was measured by western blot analysis (n = 4 per group). **E-F**. NRCFs were co-transfected with *ad-Fat10* and either a FAT10-specific E1-activated enzyme Uba6 **(E)** (n = 4 per group) interference sequence or an E2-transferase enzyme Ube2z **(F)** interference sequence; Smad3 levels in the cell extracts were measured by western blot analysis (n = 5 per group). **G** NRCFs were co-transfected with *ad-Fat10* and* sh-Ub*, Smad3 levels in the cell extracts were measured by western blot analysis (n = 3 per group). *p<0.05, **p<0.01, ***p<0.001 and ns=not significant. All data presented as mean ± SEM.

As it is known that FAT10 is a ubiquitin-like modifier which directly targets its substrates for degradation by the 26S proteasome. To confirm whether FAT10 suppresses the expression of Smad3 by promoting its degradation, NRCFs were treated with CHX, a protein synthesis inhibitor, at different times points in the presence or absence of FAT10. The data from western blotting showed that FAT10 decreased the expression of Smad3 consistent with the above data, and CHX further decreased Smad3 expression, which indicating that FAT10 modulate the degradation of Smad3 (Fig. 6A, B). Then, MG132, a proteasome inhibitor, were applied to confirm whether the activity of FAT10 on Smad3 degradation was dependent on proteasome system. As shown in Fig. 6C, treatment with CHX significantly decreased the endogenous Smad3 protein expression in NRCFs in a time-dependent manner, which was significantly inhibited by the presence of MG132. Besides, the results of Fig. 6D also confirmed that MG132 abolished the FAT10-regulated reduction in Smad3 expression. Further, RNA interference of the FAT10 specific E1-activated enzyme, UBA6, and the E2-transferase enzyme, UBE2Z were used, and the data showed that knockdown of Uba6 (Fig. 6E) or Ube2z (Fig. 6F) expression by siRNA could block FAT10 mediated degradation of Smad3. Taken together, these results suggest that FAT10 reduces Smad3 expression by promoting its proteasomal degradation.

Owing to the specific biological function of FAT10 as a ubiquitin-like modifier which not rely on secondary poly-ubiquitylation 24-27, the FAT10-proteasome system (FPS) is present. In this study, to determine whether FAT10 degraded Smad3 dependent on ubiquitin (Ub), we knocked down Ub expression in NRCFs and the data demonstrated that overexpression of FAT10 still degraded Smad3 protein in the absence of Ub (Fig. 6G). Hence, the above data indicate that the regulation of Smad3 by FAT10 is not dependent on UPS, but through FPS.

### FAT10 binds to K378 lysine residue on Smad3 directly and to influence its function

**Fig 7:**
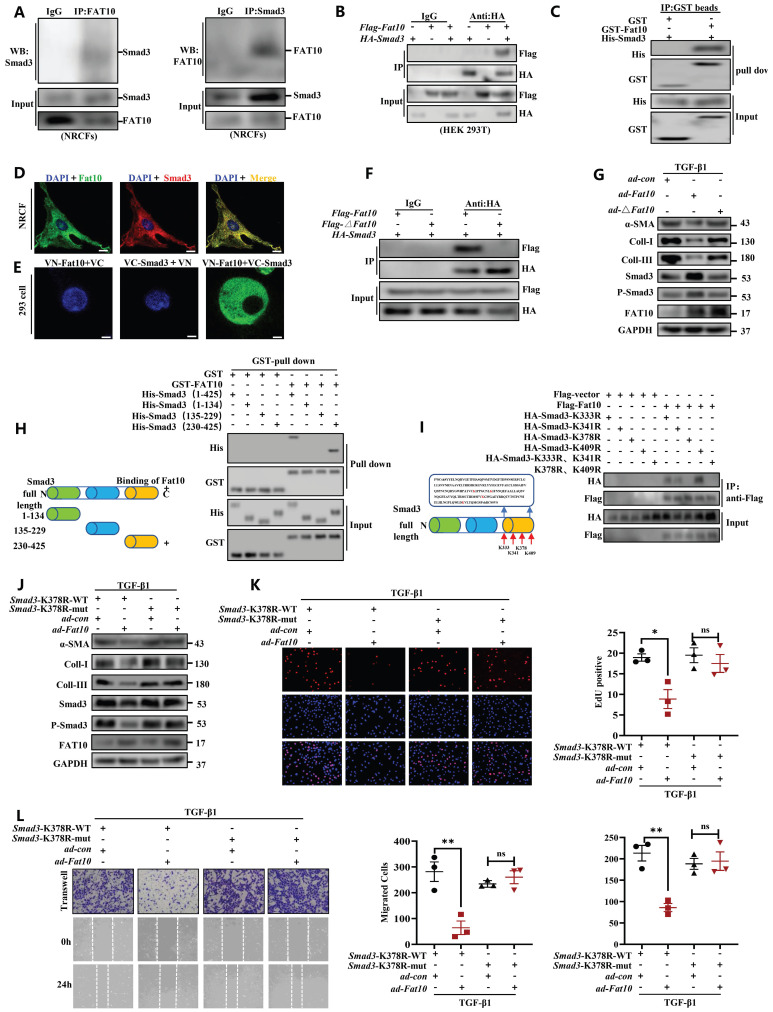
** Identification of the FAT10-binding site in Smad3. A** Co-IP for endogenous Smad3 and FAT10 in NRCFs. **B** The association between FAT10 and Smad3 in HEK293 cells expressing *Flag-Fat10* and* HA-Smad3* was detected by Co-IP.** C** Direct interaction of Smad3 with FAT10 by GST pull down assay. **D** Co-localization of Smad3 and FAT10 in NRCFs; FAT10(1:100) in red, Smad3 (1:100) in green, and DAPI nuclear counterstaining in blue. **E** Bimolecular fluorescence complementation assay exhibited interactions of FAT10 (fused with N-terminus of Venus [VN-FAT10]) with Smad3 (fused with C-terminus of Venus [VC-Smad3]. **F** Co-IP assays were performed to analyze the binding of FAT10 and Smad3 when the *Flag-△Fat10* and* HA-Smad3* were co-transfected into HEK293T cells. **G** Western Blot analysis of α-SMA, collagen I and collagen III in the NRCFs after transfection with *ad-Fat10* or *ad-△Fat10.* **H** Schematic representation of Smad3, which has three functional domains(left). Direct interaction of His-Smad3 domain and GST-FAT10 FAT by GST pull down assay(right). **I** Schematic distribution of the 4 lysine residues in domains 3 of Smad3(left). HEK293T cells were transfected with indicated constructs. Cells were lysed for Co-IP using anti-Flag beads to detect HA binding(right). **J** Western Blot analysis of in the NRCFs after transfection with Smad3-K378R-wt or Smad3-K378R-mut in the absence or presence of transfection with *ad-Fat10*.** K** The proliferation of NRCFs after transfection with Smad3-K378R-wt or Smad3-K378R-mut in the presence of transfection with *ad-Fat10* measured by EdU assays (bar 50μm).** L** The migration of NRCFs after transfection with Smad3-K378R-wt or Smad3-K378R-mut in the presence of transfection with *ad-Fat10* measured by wound-healing (bar 100μm), transwell (bar 100μm) assays. *p<0.05, **p<0.01, ***p<0.001 and ns=not significant. All data presented as mean ± SEM.

To explore whether FAT10 binds to Smad3 directly, Co-IP assay was conducted and revealed the endogenous physical interaction of Smad3 and FAT10 in NRCFs (Fig. 7A). This interaction was also confirmed in HEK293 cells when transfected with *Flag-Fat10* and *HA-Smad3* (Fig. 7B). Similar results were found by immunofluorescence assay in NRCFs and GST-pull down assay (Fig. 7C, D). Further, we performed the bimolecular fluorescence complementation assay, which is an imaging technology used to visualize protein-protein interaction within a cell. Clear fluorescent signals were exhibited when *VN-Fat10* and *VC-Smad3* expression vectors were co-transfected, further confirming that the physical interactions of FAT10 and Smad3 (Fig. 7E). All the data suggest a direct link between FAT10 and Smad3. As reported, FAT10 modulates substrate degradation via its two C-terminal glycine residues 28. Thus, we generated a plasmid and virus expressing a *Fat10* mutant lacking the two C-terminal glycine residues (*△Fat10*). As expected, the interactions between FAT10 and Smad3 were disappeared in the absence of its two C-terminal glycine residues (Fig. 7F). Moreover, unlike ad-Fat10, *ad-△Fat10* did not affect the fibrotic phenotype induced by TGF-β1 in NRCFs, as confirmed by western bloting, EdU, and transwell assays (Fig. 7G, Supplementary Fig.10). Taken together, our results provide strong evidence that FAT10 directly targets Smad3 through the C-terminal glycine residues of FAT10, which is important in inhibiting cardiac fibrosis.

Next, to identify the interaction regions of FAT10 and Smad3, three structural domains fragments of Smad3 were generated as depicted in Fig. 7H-left. GST-pull down assay demonstrated that the Smad3 region in 230-425 amino acids contains the FAT10 interaction region (Fig. 7H-right). Four lysine sites in the Smad3 binding region were mutated to arginine (230-425aa) to further confirm the specific sites binding to FAT10 (Fig. 7I-left). Co-IP experiments revealed that the binding of Smad3 to FAT10 was disappeared when the K378 site was mutated (Fig. 7I-right).

To further explore the K378 lysine binding sites of FAT10 and Smad3 in the regulation of cardiac fibrotic. NRCFs were simultaneously transiently infected with Smad3-K378R and *ad-con* or *ad-Fat10* virus. The results revealed that when co-transfected with Smad3-K378R-wt and *ad-con* or *ad-Fat10* virus, overexpression of FAT10 inhibited the function of Smad3 (Fig. 7J), fibrotic marker protein (Fig. 7J), cell proliferation (Fig. 7K) and migration (Fig. 7L). However, when Smad3 was mutated, the repression functions of FAT10 on Smad3 and fibrotic phenotype were abolished (Fig. 7J-L). In summary, our results show that FAT10 directly binds to Smad3 through the K378 site.

### Overexpression of FAT10 in hiPSC-CFs ameliorated the TGF-β1-induced fibrotic response

**Fig 8:**
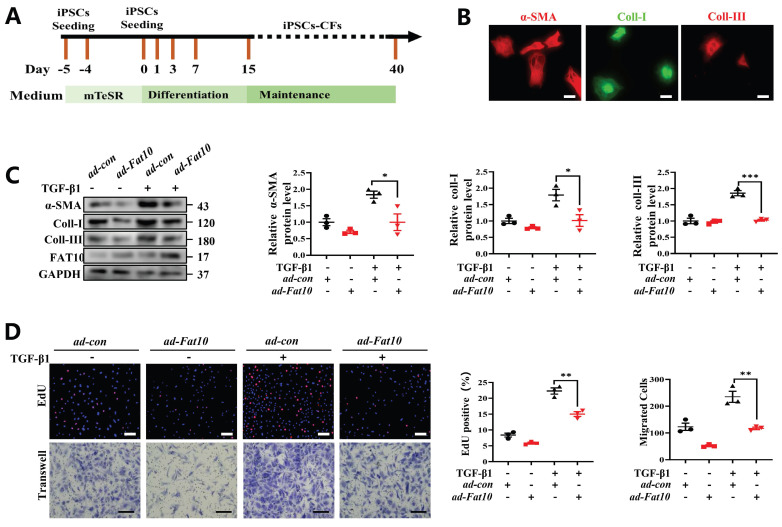
** FAT10 overexpression could ameliorate TGF-β1-induced fibrotic response in hiPSC-CFs. A** Schematic diagram of human induced pluripotent stem cell-derived cardiomyocytes (hiPSC-CFs) generated. **B** Representative immunofluorescence assays of α-SMA (left, red), collagen I (middle, green) and collagen III (right, red) (bar 50μm).** C** hiPSC-CFs were transfected with *ad-Fat10* and then subjected to treated with TGF-β1(10ng/ml), the level of α-SMA, collagen I and collagen III were detected by western blot analysis (n=3 per group). **D** Representative immunofluorescence images of EdU (bar=50μm) and migration of hiPSC-CFs measured by Transwell assay (bar 100μm) (n=3 per group). *p<0.05, **p<0.01, ***p<0.001 and ns=not significant. All data presented as mean ± SEM.

To explore whether FAT10 could regulate cardiac fibrosis in human cells, hiPSC-CFs were generated (Fig. 8A). Consistent with the above assay in vitro, hiPSC-CFs were treated with TGF-β1 to simulate a cell fibrosis model successfully which confirmed by immunofluorescence assay (Fig. 8B). As shown in Fig. 8C, the results indicated that FAT10 reversed the effects of TGF-β1-induced Increase in α-SMA, collagen I and collagen III expression in hiPSC-CFs. Meanwhile, FAT10 also suppressed TGF-β1-induced proliferation and migration of hiPSC-CFs (Fig. 8D). Above results demonstrate that FAT10 participates in TGF-β1-induced fibrosis.

## Discussion

In this study, we highlight the crucial role of FAT10 in modulation of cardiac fibrosis post-MI. To explore the function of FAT10 on cardiac fibrosis, a systemic *Fat10* knockdown (*Fat10^-/-^*) mouse was applied and *Fat10^-/-^* mice develop more severe fibrosis and functional impairment *in vivo*. Consistently, FAT10 repressed TGF-β1-induced fibrosis response in NRCFs, primary mouse CFs from *Fat10*^-/-^ mice and hiPSC-CFs. Mechanistically, the regulation of FAT10 was acted mainly dependent on increased Smad3 degradation via FPS. Notably, FAT10 targeted Smad3-K378 site directly.

Myocardial fibrosis, a common pathological change after myocardial infarction, is one of the most important reasons for irreversible ventricular remodeling. In the heart, cardiac fibroblasts (CFs) constitute the majority of non-myocyte cell instead of living myocytes. CFs trans-differentiate into myofibroblasts after MI injury and then continue to proliferate, synthesis, and secrete collagen 15, 17. Collagen forms the extracellular matrix (ECM) and plays an important role in maintaining the structural integrity of the heart and in avoiding left ventricular injury. Long-term excessive deposition of ECM reduces the compliance of the ventricle and contractile function of cardiomyocytes, leading to heart failure and increased mortality 24. Therefore, fibroblasts have become important target cells for the inhibition of myocardial fibrosis and cardiac structural remodeling. In this study, we applied NRCFs, primary CFs from *Fat10^-/-^* mouse and hiPSC-CFs to explore the regulation of myocardial fibrosis.

The initial function of FAT10, a member of UBL, is linked to cell cycle regulation, apoptosis, and immune response 8. Our team has been concerned about the role of FAT10 in cardiovascular disease, especially in MI and its complication diseases. Our team first proposed that FAT10 was expressed in the heart and played a protective role in ischemic heart disease. Moreover, we identified FAT10 exerts an important role in ischemic-induced ventricular arrhythmia. Despite it, it remains great interest for us to characterize the impact of FAT10 in cardiac fibrosis after MI. Most importantly, after MI, cardiac fibroblasts migrating into the infarct border zone, proliferating, and undergoing myofibroblast transition are common MI complications 29-31 which contribute to the repair process for maintaining cardiac integrity and function. Excessive activation of cardiac fibroblasts is a pathological state of fibrosis leading to distortion of cardiac structure and diastolic dysfunction 32. Thus, it is prudent to explore FAT10 on cardiac fibrosis.

Toward this goal, on the one hand, we employed the CRISPR/Cas9-mediated gene editing to performed *Fat10*-null mice. In our MI models of fibrosis, we demonstrated an aggravated cardiac fibrosis in *Fat10^-/-^* mice. More interesting, cardiac specific overexpression of FAT10 could reduce myocardial fibrosis and improve cardiac function, which further improve the survival rate at 28 days significantly after MI. These data suggest that FAT10 may be a key regulator of fibrosis after MI *in vivo*. On the other hand, it is well established that TGF-β1 differentiates fibroblasts into myofibroblasts and stimulates the synthesis of excessive ECM. Thus, to explore the effects of FAT10 on cardiac fibrosis *in vitro*, TGF-β1 is used as an inducer. In the present study, we demonstrated that the expression level of FAT10 is increased after TGF-β1 treatment. Our results further found that overexpression of FAT10 in CFs attenuated TGF-β1-induced myofibroblast differentiation and collagen deposition, but inhibition of FAT10 aggravated TGF-β1-induced fibrotic phenotype. Based on these findings, we proposed that FAT10 is a protective mechanism against TGF-β1-induced overactive fibrosis *in vitro*.

Then, an IP-MS assay was performed to make sure the underlying mechanisms of FAT10 on fibrosis, and the data supported that Smad3 may be the downstream molecular of FAT10 in cardiac fibrosis. Smad3, a member of Smads family, has been reported to be essential for the development of cardiac fibrosis 17, 18. To verify the involvement of Smad3 in FAT10-mediated myocardial fibrosis, we first examined the effects of FAT10 on Smad3 expression. As expected, western blotting assay concluded that FAT10 decreased Smad3 expressions. Previous work has figured out that deletion of Smad3 could markedly inhibit collagen deposition in the scar, and attenuated dilative remodeling and diastolic dysfunction after MI 16. In addition, Smad3 knockdown prevents Ang-II induced hypertensive cardiovascular 19. Studies have also demonstrated Smad3 gene disruption could directly attenuated TGF-β1-mediated fibroblast responses. Consistent with others, with the help of pharmacological drug, our further work demonstrated that FAT10 modulated fibrosis via Smad3 *in vivo* and *in vitro*.

Third, by utilizing the protein synthesis inhibitor CHX and the proteasome inhibitor MG132, we found that FAT10 promotes the degradation of Smad3. Then, IP and GST-pull down experiments showed a direct interaction between FAT10 and Smad3. Furthermore, a C-terminal glycine-deficient FAT10 mutant plasmid was constructed and IP assays verified that FAT10 bound to Smad3 via its C-terminal diglycine motif consistent with previous data. Additionally, three structural domains fragments of Smad3 plasmids were generated and used to explore the specific binding sites of Smad3 binding with FAT10. Finally, both the ΔFat10 plasmid and Smad3^K378R^ failed to suppress fibrosis in NRCMs under TGF-β stimulation. To our knowledge, this is the first study to indicate that Smads family protein are substrates of FAT10 and clarify the specific role and mechanism of FAT10 in regulating Smad3.

FAT10 is the unique member of ubiquitin-like protein family which contains two tandem ubiquitin-like domains and efficiently mediates protein degradation in a proteasome system-dependent manner 33. However, our previous studies showed different role of FAT10 on its substrates. Reportedly, FAT10 stabilizes β-catenin and Cavoulin3 expression by antagonizing substrate ubiquitination 9, 10, 12. Moreover, FAT10 is found to antagonize SIRT1 SUMOylation to decrease its expression and activity by blocking the formation of SUMO1-SIRT1 complexes 10. Additionally, FAT10 binds to the C-terminal fragments of Nav1.5, stabilizing Nav1.5 expression upon prevention of its Neddylation and degradation 12. In this study, an interesting finding of FAT10 in Smad3 regulation be independent on ubiquitin-mediated PS, but through FPS is strongly confirmed.

Given the complexity in regulation of fibrosis response, several important questions remain to be answered in the future. First, although we tried to use a variety of methodologies to confirm the role of FAT10 in myocardial fibrosis after MI, it would be more reliable if cardiac fibroblasts specific knockout and overexpression mice were used. Furthermore, the clinical value of FAT10 as biomarker for cardiac fibrosis after MI remains to be further studied in MI patients in the future.

These findings provide the first evidence of a protective role of FAT10 in ischemic-induced cardiac fibrosis. FAT10 attenuated cardiac fibrosis post MI *in vivo* and repressed TGF-β1-induced fibrosis response in NRCFs, primary mouse CFs from *Fat10^-/-^* mice and hiPSC-CFs *in vitro*. Mechanistically, FAT10 binds to Smad3-K378 site directly via its C-terminal glycine residues and increased Smad3 degradation via FPS. Altogether, these findings suggest that enhancing FAT10 maybe a novel therapeutic strategy to prevent/reverse cardiac fibrosis after MI.

## Supplementary Material

Supplementary methods, figures and tables.
