# Supplementary material for: Ubiquitin like protein FAT10 repressed cardiac fibrosis after myocardial ischemic via mediating degradation of Smad3 dependent on FAT10-proteasome system
Source: Int J Biol Sci. 2023 Jan 16;19(3):881–96. doi: 10.7150/ijbs.77677 (PMC9910007; doi:10.7150/ijbs.77677)
Supplement: Supplementary file 1 — Supplementary methods, figures and tables. [file ijbsv19p0881s1.pdf]

Supplementary material for

Ubiquitin like protein FAT10 repressed cardiac fibrosis after myocardial ischemic via mediating degradation of Smad3 dependent on FAT10-proteasome system

Running title: FAT10 and cardiac fibrosis

Chen Chen[1,2]* • Xiaoqing Li[1,2]* • Tao Zhou[2*,] • Yuhao Su[1,2] • Bodong Yu[4] • Jiejing Jin[2] • Jinyan Xie[2] • Yang Shen[2,3] • Rong Wan[2#] • Kui Hong[1,2,3#]

[1] Department of Cardiovascular Medicine, the Second Affiliated Hospital of Nanchang University, Nanchang of Jiangxi, 330006 China; [2] Jiangxi Key Laboratory of Molecular Medicine, Nanchang of Jiangxi, 330006 China; [3] Department of Genetic Medicine, the Second Affiliated Hospital of Nanchang University, Nanchang of Jiangxi, 330006 China; [4] Second College of Clinical Medicine, Nanchang University, Nanchang, Jiangxi, 330006 China

*These authors contributed equally to the manuscript

# Address for correspondence:

Rong Wan, PhD

Jiangxi Key Laboratory of Molecular Medicine,

The Second Affiliated Hospital of Nanchang University, Nanchang of Jiangxi, 330006, China

Email: rong87223@163.com

Kui Hong, MD, PhD

Department of Cardiovascular Medicine and Jiangxi Key Laboratory of Molecular Medicine,

The Second Affiliated Hospital of Nanchang University, Nanchang of Jiangxi, 330006, China

Email: hongkui88@163.com

1. Supplemental Methods

1.1 Masson, Sirius Red, Immunochemistry and Immunofluorescence staining

Heart sections were prepared at 4-μm thickness through a routine procedure. The sections were subsequently stained with Masson trichrome and Sirius Red staining to determine the extent of fibrosis[1]. For immunochemistry and immunofluorescence staining (IHC), the sections were incubated with primary antibodies which listed in **Supplementary Table 1.**

1.2 Ischemic animal model and surgical procedure

Myocardial infarction (MI) model was created in *Fat10*$^{-/-}$ and WT mice via ligation of the left anterior descending artery (LAD). Regional ischemia of the heart was confirmed by ECG, which showed ST elevation. Control group underwent sham operations without ligation of the LAD.

1.3 Echocardiography

In Briefly, mice were anesthetized with 1% isoflurane at a rate of 1.0%/min. Using the Canadian Visualsonics Vevo 2100 color ultrasound diagnostic apparatus, equipped with a 17MHz transducer, scanning the left sternal border of the mouse, the right sternal border, the apex and the sternal fossa, collecting ultrasound images of the heart, and performing cardiac structures, each Measurement of valve blood flow and time parameters.

1.4 Adenovirus and plasmid constructs

All these recombinant adenoviruses (*ad-Fat10, sh-Fat10, ad-Smad3, sh-Smad3, sh-Ub, Smad3-K378R-wt, Smad3-K378R-mut*) were designed by Gene-Chen (Shanghai Co., Ltd). The Flag-*Fat10*, GST-*Fat10*, deletion forms of Smad3(1-134, 135-229, 230-425 amino acids (a.a)), mutant forms Smad3(K333R, K341R, K378R, K409R) (lyshne mutated to arginine) were also established from

Gene-Chen (Shanghai Co., Ltd). *Fat10* C-terminal diglycine motif-deficient plasmid (△*Fat10*), and related adenovirus, *ad-Fat10*, *sh-Fat10*, *ad-Smad3* and *sh-Smad3* adenovirus were obtained from Gene Chem Company (*Shanghai, China*). UBE2Z, and UB6A interference fragment kits were purchased from GenePharma Company (*Shanghai, China*).

1.5 Isolation of primary cardiac fibroblasts

Firstly, the hearts were placed in a 10cm dish with cold Dulbecco's modified Eagle's medium/Low glucose (DMEM, Hyclone, Logan, Utah, USA), and cut to approximately 1 mm3 with scissors. The heart tissues were digested in 0.25% trypsin with gently shaking, all digestive fluid was collected in DMEM with 10% fetal bovine serum (FBS, Sciencell, San Diego, California, USA) and penicillin (100 IU/ml) and streptomycin (100 mg/ml). Then the cells were filtered through 75 μm cell strainer and centrifuged at 1200 rpm for 5 min. Finally, the isolated cardiac cells were seeded in six-well plates for 90 min, the pre-seeding medium containing CM were removed and seeded in new six-well plates, while the CF which could be adhere to the culture plates. All CFs in this study were treated within three passage cultures.

1.6 Western blotting (WB), co-immunoprecipitation Co-IP, Immunofluorescent (IF) and GST pull-down assays

WB, Co-IP, IF and GST pull-down assays were performed as previously described[2, 3]. All antibodies listed in **Supplementary Table 1.**

1.7 Cell proliferation and Migration

Edu cell proliferation kit (RiboBio, Guangzhou, China) was used to measure cells proliferation. Wound Healing Assay, transwell assay were used to detect cell migration as describe previously[4].

Supplementary Tables

Supplementary Table 1. The baseline of cardiac function of wild-type and Fat10-/- mice measured by echocardiography

Cardiac function	*Wild-type*(n=6)	*Fat10*$^{-/-}$(n=6)	P
LVVol;d	36.87±1.73	37.3±1.51	0.66
LVVol;s	13.03±0.45	12.94±0.60	0.78
IVSd(mm)	0.71±0.06	0.70±0.05	0.95
IVSs(mm)	0.84±0.05	0.81±0.03	0.70
LVIDd(mm)	3.07±0.09	3.05±0.06	0.91
LVIDs(mm)	1.83±0.08	1.87±0.08	0.39
LVPWd(mm)	0.91±0.08	0.88±0.12	0.59
LVPWs(mm)	1.06±0.12	1.00±0.01	0.44
LVVol;d: left ventricular volume at diastolic phase; LVVol;s: left ventricular volume at systolic phase; SV: stroke volume; LVIDd: left ventricular internal diameter at diastolic phase; LVIDs: left ventricular internal diameter at systolic phase; IVSd: interventricular septal thickness at diastolic phase; IVSs: interventricular septal thickness at systolic phase; LVPWd: left ventricular posterior wall thickness at diastolic phase; LVPWs: left ventricular posterior wall thickness at systolic phase.			

Supplementary Table 2. The following antibodies were used for western blotting and immunostaining

Target protein	Source (Catalog number)
FAT10	Abcam (ab168680)
Smad3	Abcam (ab40854)
Smad3	66516-1-Ig
UBD Fushon Protein	Proteintech (Ag27706)
Recombinant Human Smad3 protein	Abcam (ab89353)
Smooth Muscle Actin	Proteintech (14395-1-AP)
Collagen Type I	Proteintech (14695-1-AP)
Collagen Type III	Proteintech (22734-1-AP)
p-Smad3	Abcam (ab52903)
Smad2	Abcam (ab40855)
p-Smad2	Abcam (ab280888)
Smad4	Abcam (EP618Y)
Smad7	Proteintech (25840-1-AP)
GST	Proteintech (10000-0-AP)
Flag	Proteintech (66008-2-Ig)
HA	Proteintech (51064-2-AP)
His	Proteintech (66005-1-Ig)
GAPDH	Proteintech (60004-1-Ig)
MG132	Selleck (S2619)
CHX	Selleck (S3648)
TNF-α/IFN-γ	PeproTech

Supplemental Figures

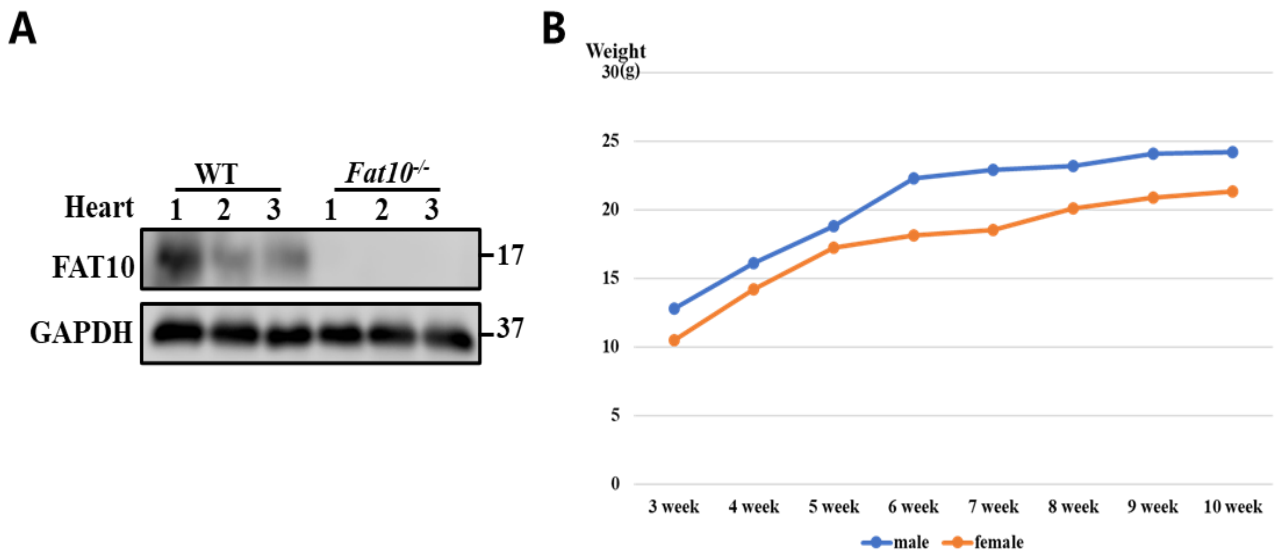

Supplemental Figure S1. Validation and basic information of *Fat10*$^{-/-}$ mice. **(A)**Western blotting was performed to detect the expression of FAT10 in heart tissues of WT and *Fat10*$^{-/-}$ mice. **(B)** Growth weight curves of *Fat10*$^{-/-}$ mice.

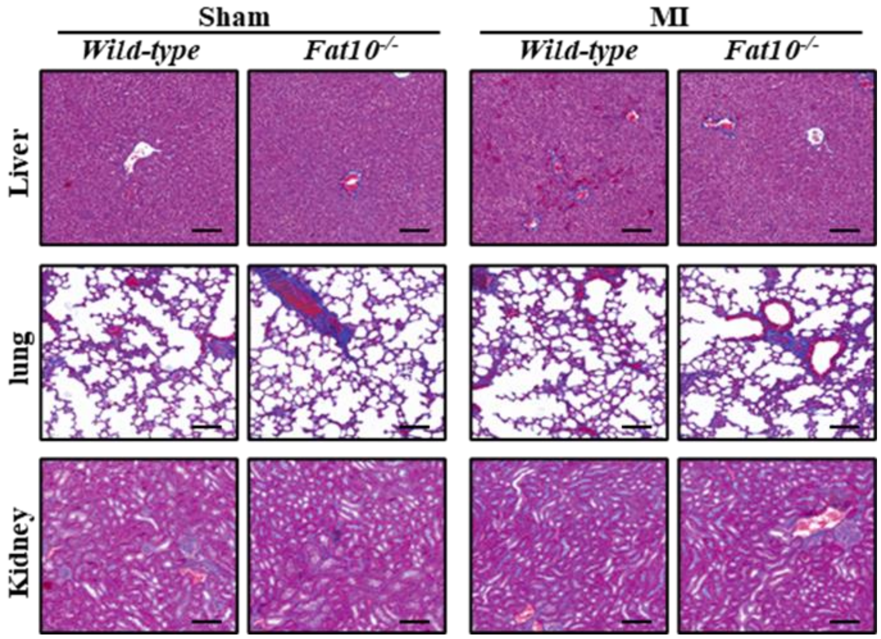

Supplemental Figure S2. Masson staining of liver, lung and kidney in each group.

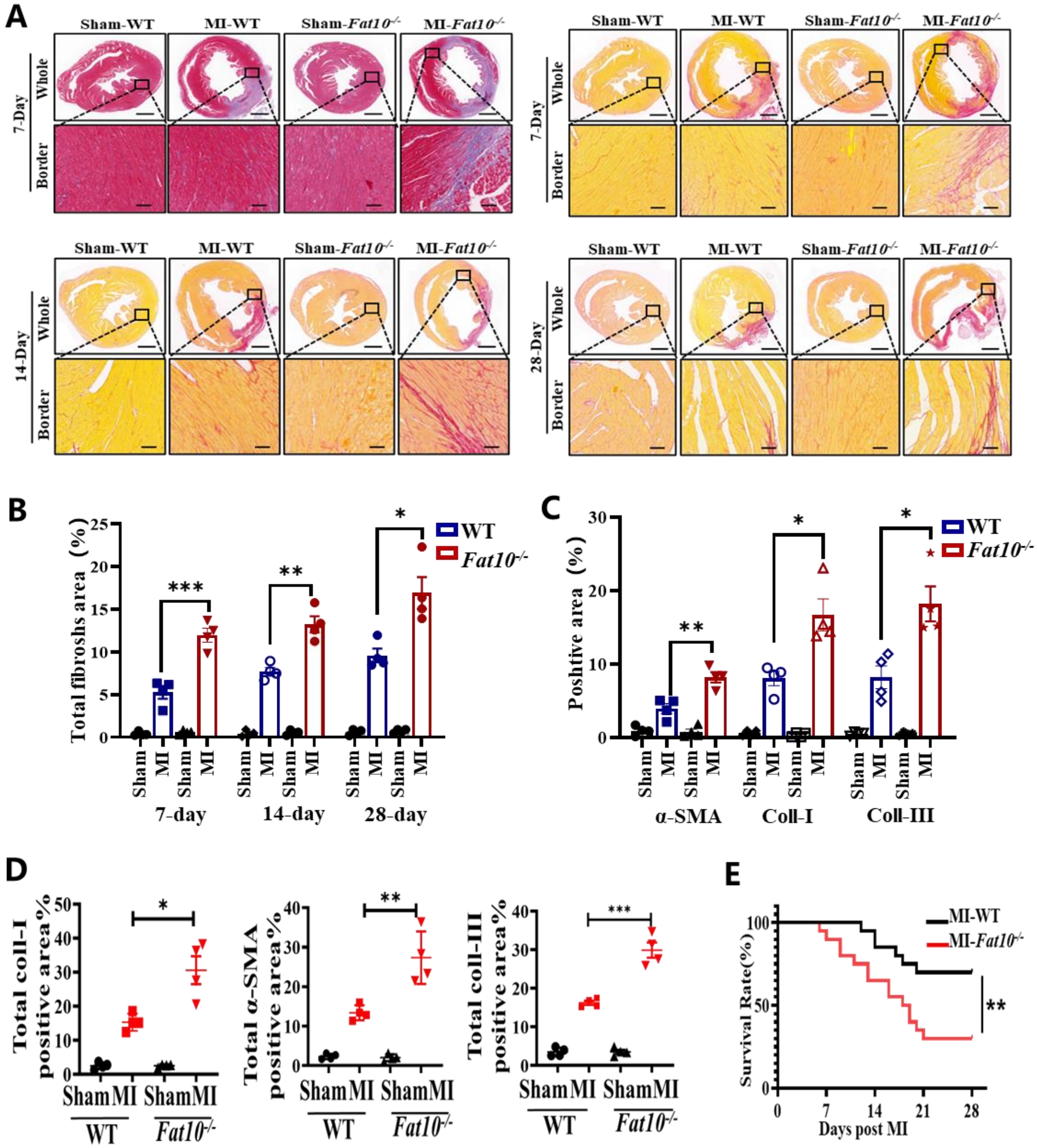

Supplemental Figure S3. FAT10 deficiency aggravates MI-induced cardiac fibrosis (A) Masson and Sirius Red staining of whole heart fibrosis (up, bar 1mm) and infarct border fibrosis (down, bar 50μm). **(B)** Quantification of the total fibrotic area by Masson staining in 7, 14, 28day after being subject MI operation. **(C)** Quantification of α-SMA, collagen I and collagen III positive area by Immunofluoresence staining from WT and Fat10$^{-/-}$ mice after being subject 14day-sham operation (n=4 per group). **(D)** Quantification of α-SMA, collagen I and collagen III positive area by IHC from WT and Fat10$^{-/-}$ mice after being subject 14day-sham operation **(E)** Kaplan-Meier survival curves of mice in the 28 days after MI (n=20 per group). *p＜0.05, **p<0.01, ***p<0.001 and ns=not significant. All data presented as mean±SEM.

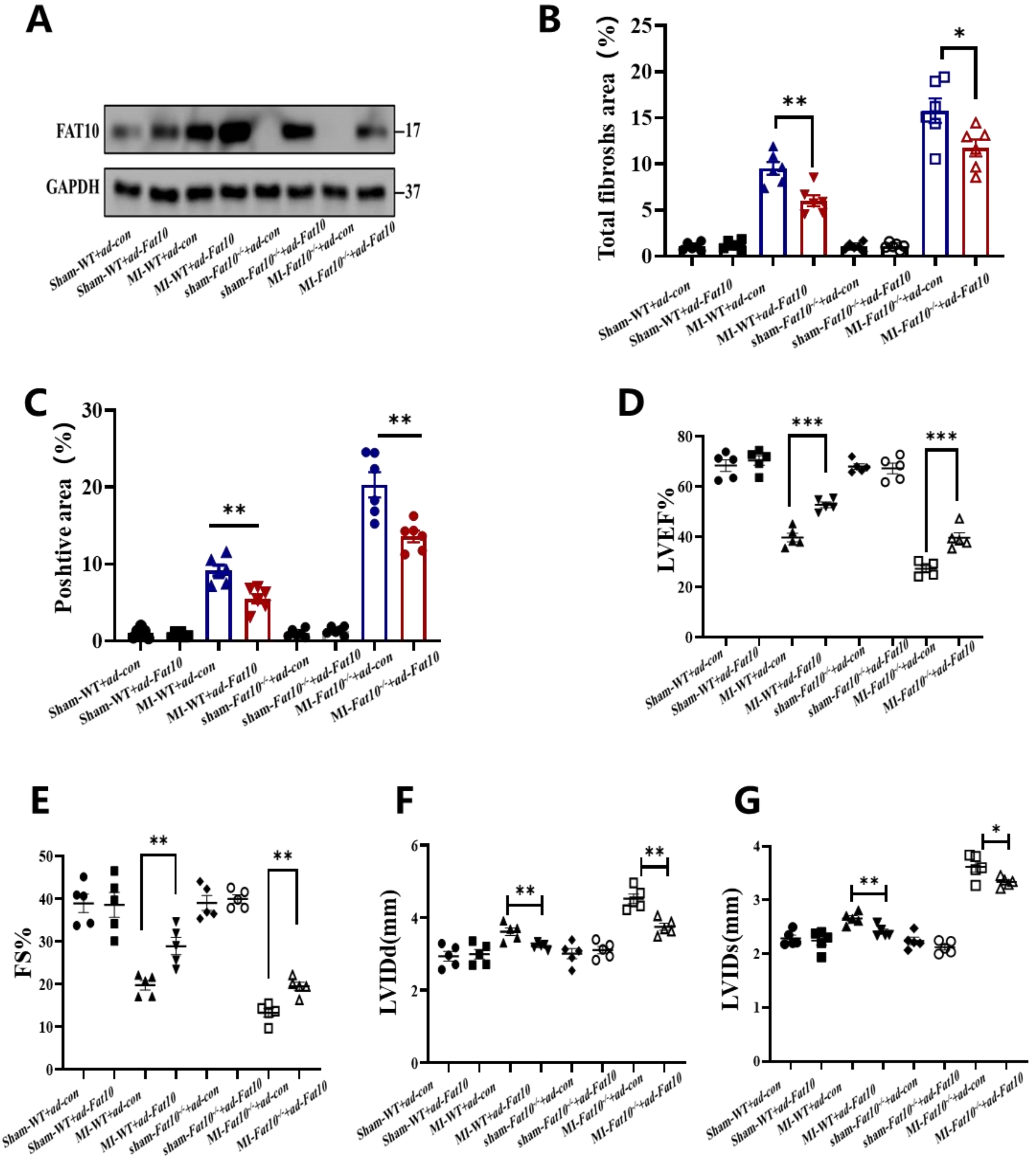

Supplemental Figure S4. Overexpression of FAT10 alleviates the MI-induced cardiac fibrosis. (A) Western blotting analyses of FAT10 expression in per group. **(B)** Quantification of the total fibrotic area by Masson staining in per group. **(C)** Quantification of collagen I by Immunofluoresence staining in per group. **(D-G)** Echocardiographic analysis to evaluate left ventricular ejection fraction (LVEF), fractional shortening (FS), left ventricular internal diameter at end-diastole (LVIDd) and left ventricular internal diameter at end-systole (LVIDs) in WT and Fat10-/- after sham and 2 weeks post-MI surgery. *p $<$ 0.05, **p<0.01, ***p<0.001 and ns=not significant. All data presented as mean ± SEM; comparison by unpaired t-test.

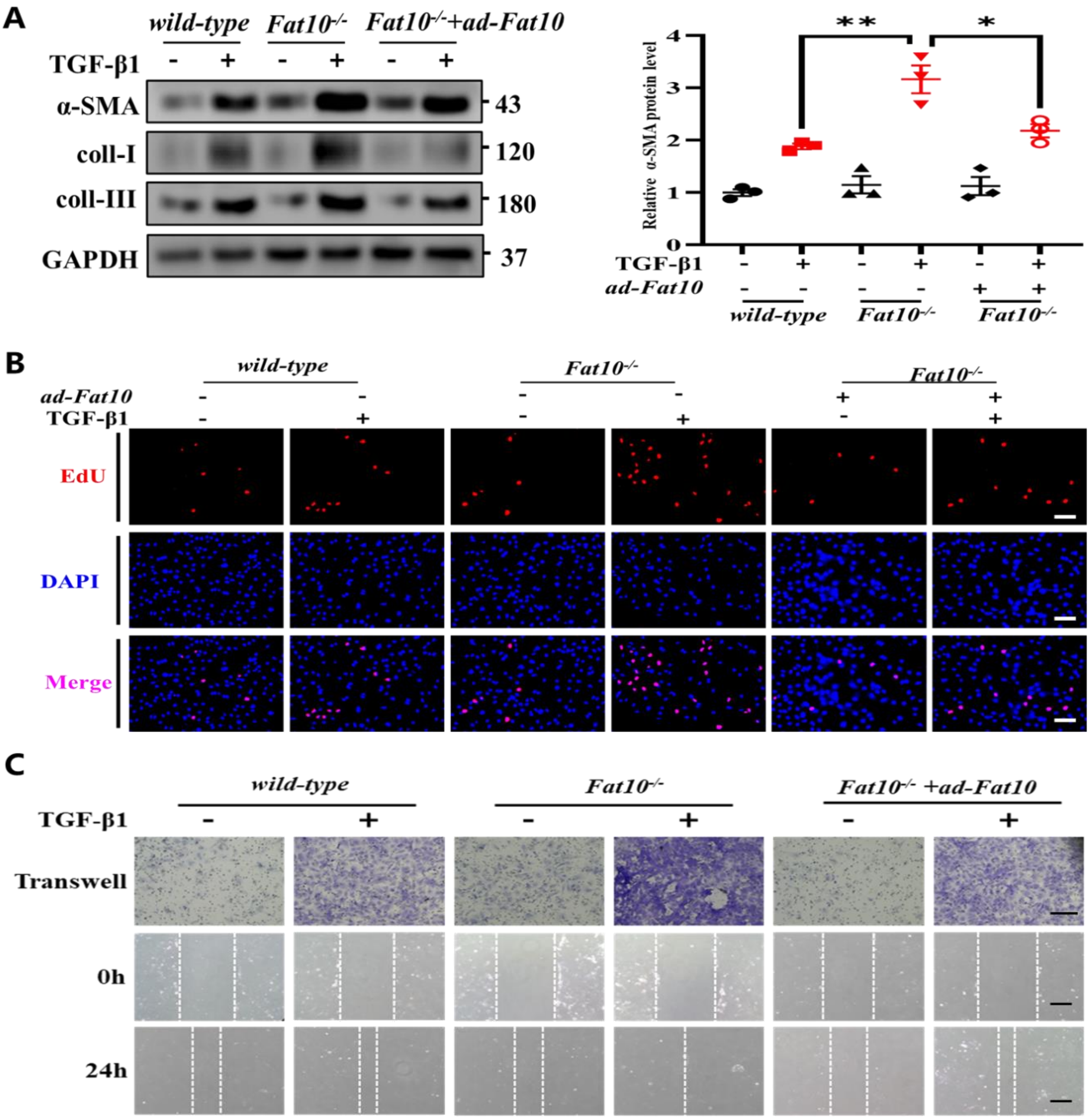

Supplemental Figure S5. FAT10 inhibits the TGF-β1-induced fibrotic response in primary mouse CFs. Primary mouse CFs from the LV of WT mice and *Fat10*$^{-/-}$ mice and treated with TGF-β1. **(A)** The level of α-SMA, collagen I and collagen III were detected by western blotting analysis. **(B)** Representative immunofluorescence images of EdU(red) and DAPI (blue)(left) (right) in the different experimental groups (bar 50μm). **(C)** Migration measured by transwell assay (bar 100μm) and wound-healing assay (bar 100μm). *p＜0.05, **p<0.01, ***p<0.001 and ns=not significant. All data presented as mean±SEM.

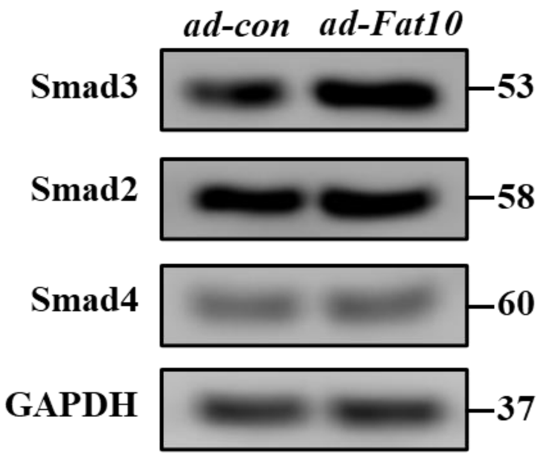

Supplemental Figure S6. FAT10 did not affect Smad2 and Smad4 protein expresshon. Western blotting analysis of Smad2, and Smad4 expression.

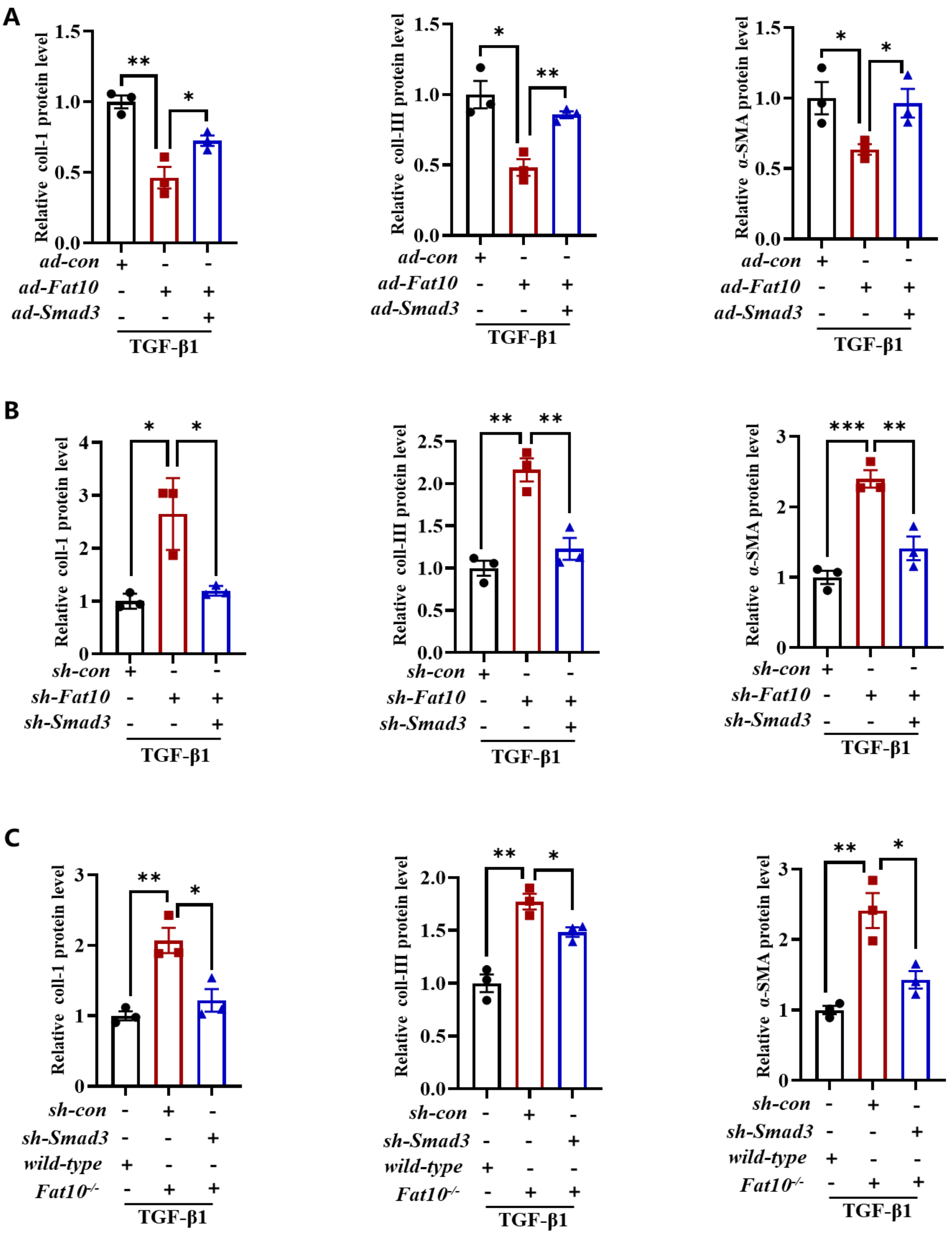

Supplemental Figure S7. FAT10 regulates the expressions of cardiac fibrotic marker by affecting Smad3. All group were transfected with adenovirus and then exposed to TGF-β1. **(A)** Quantitative assessment of the protein level of collagen I, collagen III and α-SMA, NRCFs were co-transfected with *ad-Fat10* and *ad-Smad3* or *ad-con* (n=3 per group). **(B)** Quantitative assessment of the protein level of collagen I, collagen III and α-SMA, NRCFs were co-transfected with *sh-Fat10* and *sh-Smad3* or *sh-con* (n=3 per group). **(C)** Quantitative assessment of the protein level of collagen I, collagen III and α-SMA, *Fat10*$^{-/-}$ and WT primary CF were transfected with *sh-Smad3* (n=3 per group). *p＜0.05, **p<0.01, ***p<0.001 and ns=not significant. All data presented as mean±SEM.

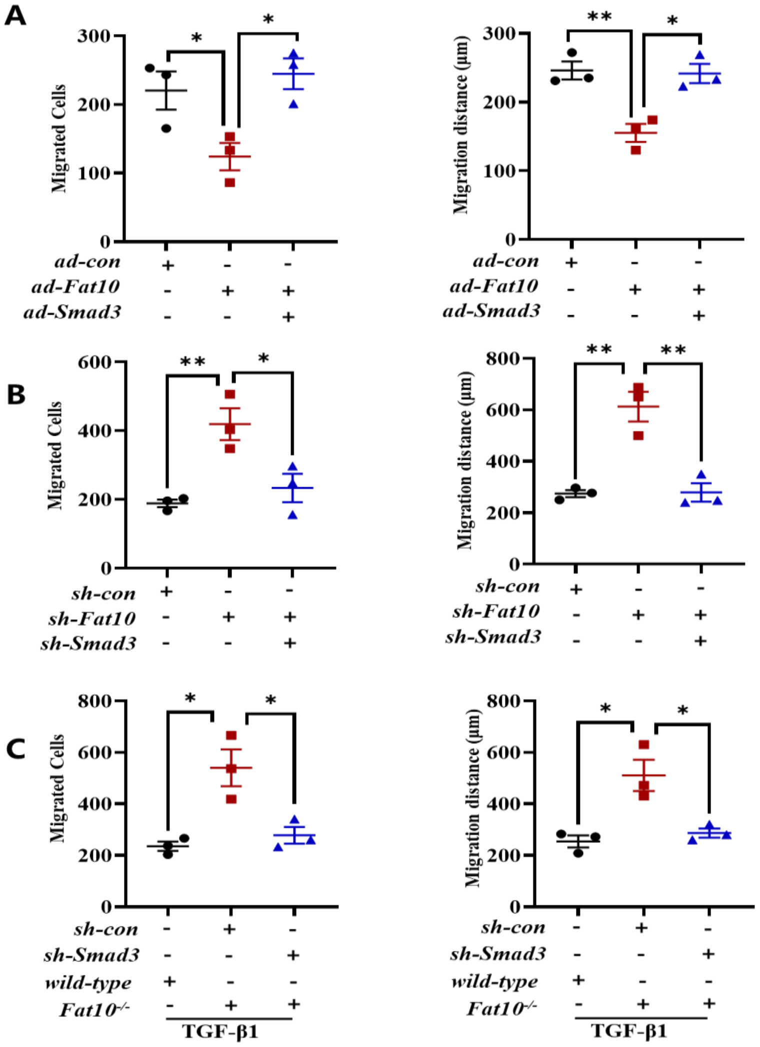

Supplemental Figure S8. FAT10 regulates the CF migration by affecting Smad3. All group were transfected with adenovirus and then exposed to TGF-β1. **(A)** The quantitative data of CF migration by transwell assay (left) and wound-healing assay (right), NRCFs were co-transfected with *ad-Fat10* and *ad-Smad3* or *ad-con* (n=3 per group). **(B)** The quantitative data of CF migration by Transwell assay (left) and wound-healing assay (right), NRCFs were co-transfected with *sh-Fat10* and *sh-Smad3* or *sh-con* (n=3 per group). **(C)** The quantitative data of CF migration by transwell assay (left) and wound-healing assay (right), *Fat10*$^{-/-}$ and WT primary CF were transfected with *sh-Smad3* (n=3 per group). *p＜0.05, **p<0.01, ***p<0.001 and ns=not significant. All data presented as mean±SEM.

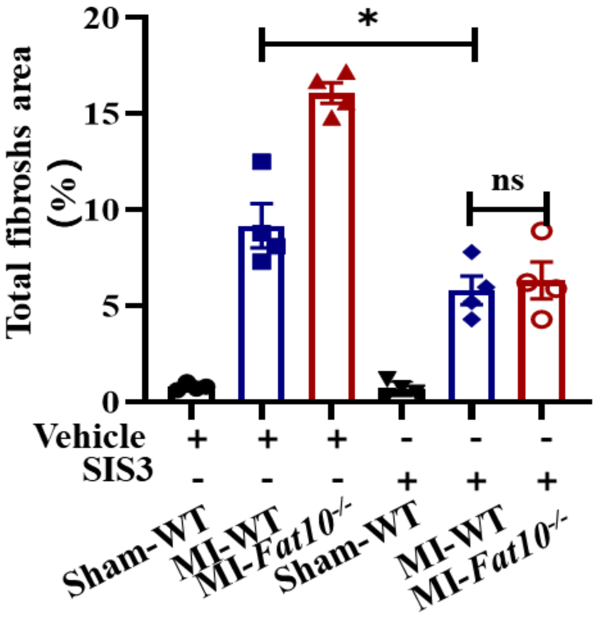

Supplemental Figure S9. Quantification of the total fibrotic area by Masson staining in per group.

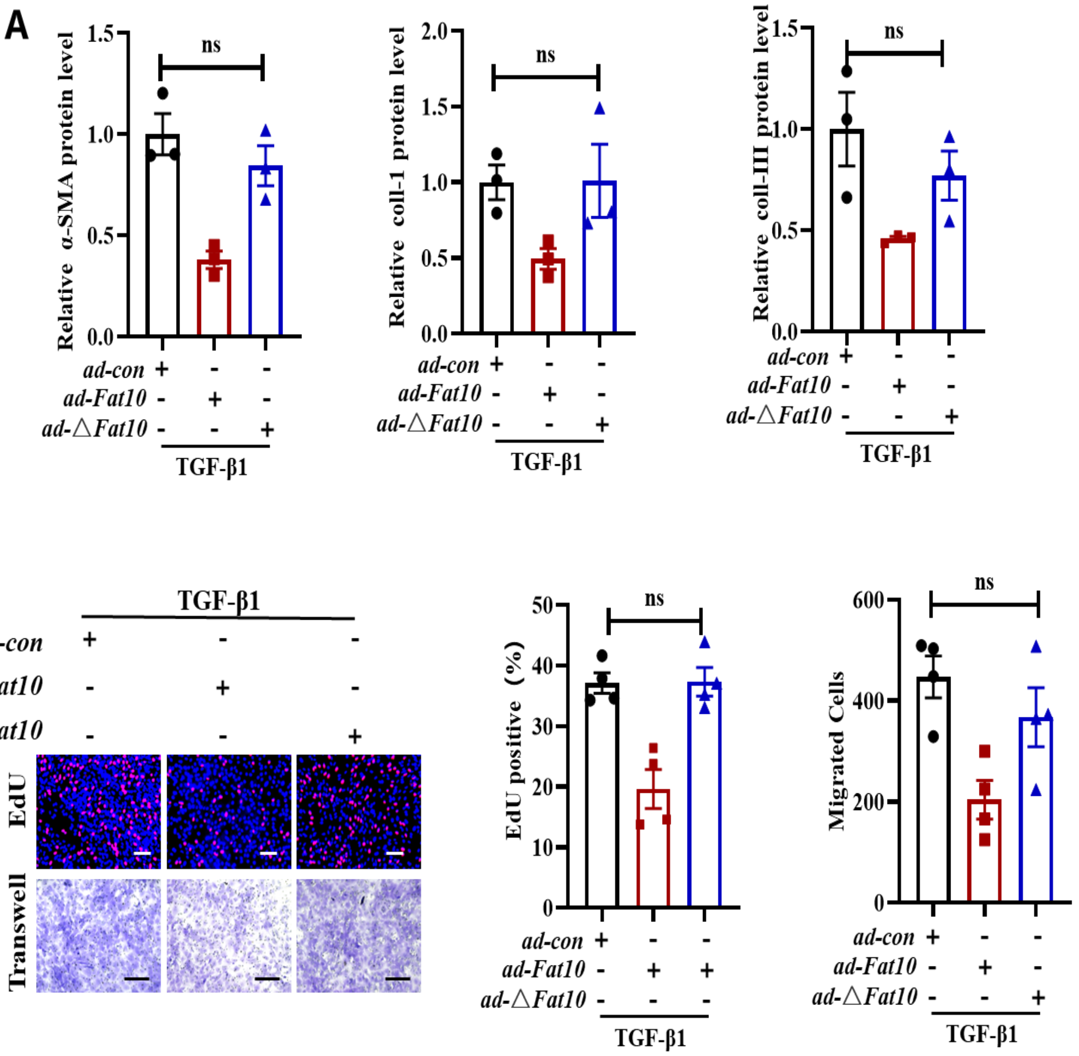

Supplemental Figure S10. FAT10 regulates the cardiac fibrotic marker via the K378 site of Smad3. All group were transfected with adenovirus and then exposed to TGF-β1. **(A)** Quantitative assessment of the protein level of α-SMA, collagen and collagen III when NRCFs after transfection with *ad-Fat10* or *ad-△Fat10* (n=3 per group). **(B)** Proliferation measured by EdU assay (up) and migration measured by transwell assay (down) (n=3 per group).

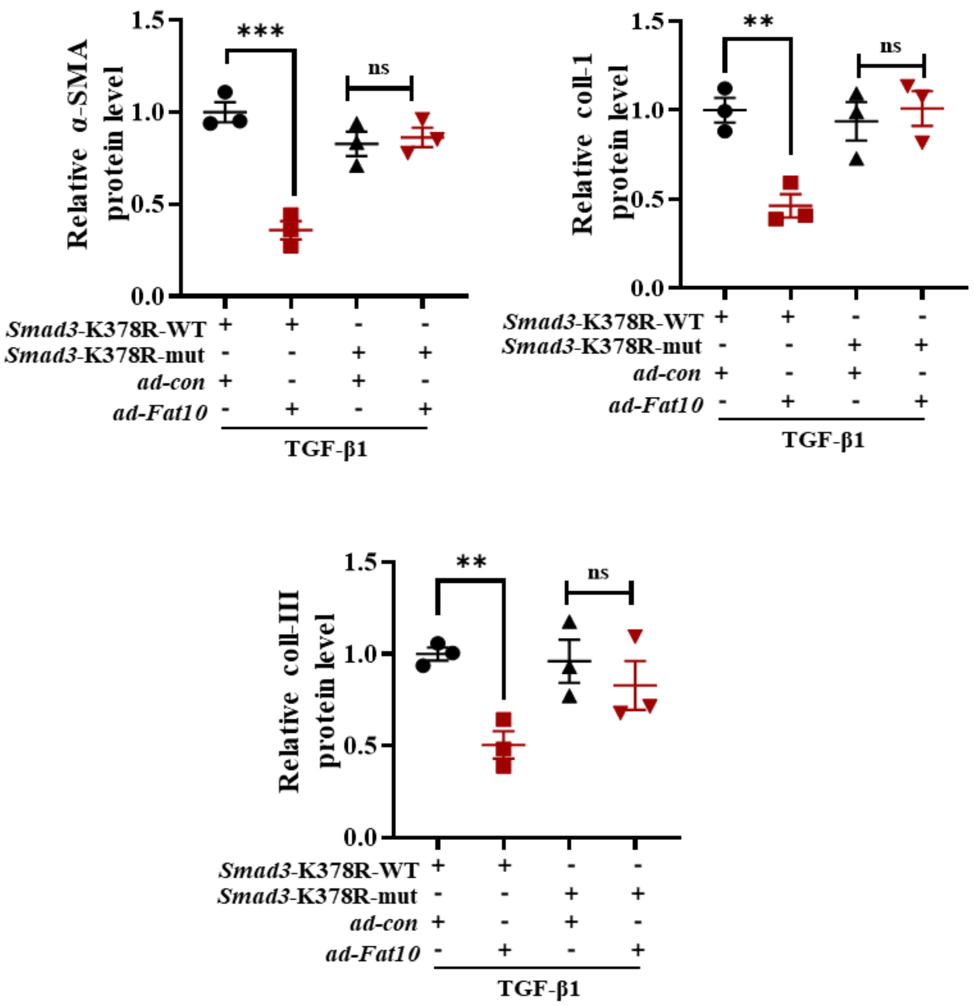

Supplemental Figure S11. Quantitative assessment of the protein level of α-SMA, collagen and collagen III when NRCFs after transfection with Smad3-K378R-wt or Smad3-K378R-mut in the presence of transfection with *ad-Fat10* (n=3 per group). *p＜0.05, **p<0.01, ***p<0.001 and ns=not significant. All data presented as mean±SEM.

References:

1. Nagai T, Honda S, Sugano Y, Matsuyama TA, Ohta-Ogo K, Asaumi Y, Ikeda Y, Kusano K, Ishihara M, Yasuda S, Ogawa H, Ishibashi-Ueda H, Anzai T. Decreased myocardial dendritic cells is associated with impaired reparative fibrosis and development of cardiac rupture after myocardial infarction in humans. *J Am Heart Assoc* 2014;**3**(3):e839.
2. Zhou Q, Peng X, Liu X, Chen L, Xiong Q, Shen Y, Xie J, Xu Z, Huang L, Hu J, Wan R, Hong K. FAT10 attenuates hypoxia-induced cardiomyocyte apoptosis by stabilizing caveolin-3. *J Mol Cell Cardiol* 2018;**116**:115-124.
3. Liu X, Ge J, Chen C, Shen Y, Xie J, Zhu X, Liu M, Hu J, Chen L, Guo L, Zhou Q, Yan X, Qiu Y, Wan R, Marian AJ, Hong K. FAT10 protects against ischemia-induced ventricular arrhythmia by decreasing Nedd4-2/Nav1.5 complex formation. *Cell Death Dis* 2021;**12**(1):25.
4. Liu Z, Xu Q, Yang Q, Cao J, Wu C, Peng H, Zhang X, Chen J, Cheng G, Wu Y, Shi R, Zhang G. Vascular peroxidase 1 is a novel regulator of cardiac fibrosis after myocardial infarction. *Redox Biol* 2019;**22**:101151.
